# Angiogenic Transformation in Human Brain Micro Endothelial Cells: Whole Genome DNA Methylation and Transcriptomic Analysis

**DOI:** 10.3389/fphys.2019.01502

**Published:** 2019-12-11

**Authors:** Dipali Goyal, Ravi Goyal

**Affiliations:** School of Animal and Comparative Biomedical Sciences, University of Arizona, Tucson, AZ, United States

**Keywords:** epigenetic, vasculogenesis, arteriogenesis, RNA-seq, angiogenic differentiation

## Abstract

We tested the hypothesis that endothelial capillary tube formation in 3D cultures in basement membrane extract (BME) is secondary to the altered DNA promoter methylation and mRNA expression in human brain micro endothelial cells (HBMECs). We conducted a whole-genome transcriptomic and methylation microarray and CRISPR/Cas9-mediated gene knockdown to test our hypothesis. The data demonstrated that with angiogenic transformation 1318 and 1490 genes were significantly (*p* < 0.05) upregulated and downregulated, respectively. We compared our gene expression data with the published databases on GEO and found several genes in common. PTGS2, SELE, ID2, HSPA6, DLX2, HEY2, FOSB, SMAD6, SMAD7, and SMAD9 showed a very high level of expression during capillary tube formation. Among downregulated gene were ITGB4, TNNT1, PRSS35, TXNIP, IGFBP5. The most affected canonical pathways were ATM signaling and cell cycle G2/M DNA damage checkpoint regulation. The top upstream regulators of angiogenic transformation were identified to be VEGF, TP53, HGF, ESR1, and CDKN1A. We compared the changes in gene expression with the change in gene methylation and found hypomethylation of the CpG sites was associated with upregulation of 515 genes and hypermethylation was associated with the downregulation of 31 genes. Furthermore, the silencing of FOSB, FZD7, HEY2, HSPA6, NR4A3, SELE, PTGS2, SMAD6, SMAD7, and SMAD9 significantly inhibited angiogenic transformation as well as cell migration of HBMECs. We conclude that the angiogenic transformation is associated with altered DNA methylation and gene expression changes.

## Introduction

Cerebral angiogenesis is an essential mechanism for restoring cerebral perfusion under various physiological and pathological conditions. For instance, autopsy studies have demonstrated that increased angiogenesis in the ischemic brain region following stroke correlated with increased patient survival (Krupiński et al., 1993). In fact, the major cause of disability following stroke is caused by the lack of the angiogenic response (Basnyat, 2005; Mozaffarian et al., 2015). Moreover, as we grow old the angiogenic potential decreases. However, little is known about the molecular identities of the pathways involved in cerebral angiogenesis. Thus, a complete understanding of angiogenic pathways is critical for the development of new pharmacologic tools and strategies for the management of conditions with dysregulated cerebral perfusion.

Previous studies have analyzed gene expression changes as a consequence of *in vitro* capillary tube formation in human umbilical vein endothelial cells (HUVEC). However, to our knowledge, there were no such studies conducted on endothelial cells obtained from the adult human brain. Of importance, the biological characteristics of different vascular beds are significantly different (Blitzer et al., 1996). For instance, under hypoxic conditions, pulmonary bed demonstrate significant vasoconstriction (Wang et al., 2012; Grimmer and Kuebler, 2017), whereas cerebral circulation demonstrates significant vasodilation (Blitzer et al., 1996). Thus, we conducted the present study on human brain microendothelial cells. Additionally, we provide a comparative analysis of the changes in gene expression in HUVEC versus human brain micro endothelial cell (HBMEC).

There are various “models” to study angiogenesis such as *in vitro* basement membrane extract (BME) 3-dimensional culture model, *in vitro* aortic ring assay, *in vivo* BME plug assay in a mouse, chick chorioallantoic membrane assay, corneal neovascularization assay, and so forth (Auerbach et al., 2003). To elucidate the pathways involved in angiogenesis, we decided to examine the gene expression changes in HBMECs with angiogenic transformation in 3-D cultures in BME. This is regarded as one of the most specific tests and is reasonably close to the *in vivo* situations (Madri et al., 1988). Moreover, 3D culture with BME has been demonstrated to induce capillary formation with a proper lumen by electron microscopy studies (Connolly et al., 2002).

One of the major epigenetic mechanism by which gene expression is regulated is by alteration in the methylation of cytosine nucleotides in the gene promoter regions. Cytosine methylation alters the DNA hydrophobic characteristics and inhibits the binding of transcription activators or suppressors. Chiefly, promoter DNA methylation is associated with reduced transcription, and an increase in DNA methylation in the gene body is associated with increased transcription (Yang et al., 2014). The increase in transcription is achieved by inhibiting the binding of transcription activators, or by increased binding of methyl-CPG proteins with repressive chromatin remodeling activities. Because of the requirement for coordinated transcriptional changes during angiogenesis, there is a significant alteration in DNA methylation/demethylation during this process. However, the role of DNA methylation and the resulting transcriptional control of gene expression during angiogenesis are not completely known. The present study is an attempt to elucidate some of the mechanistic events involved during this complicated process.

## Materials and Methods

We conducted the experiments on human primary brain microendothelial cells obtained from the Cell Systems Repository of Applied Cell Biology Research Institute (Kirkland, WA, United States). We performed the experiments cells at passage 8. We confirmed and characterized the cells as described previously (Mata-Greenwood et al., 2017). Briefly, we confirmed cell morphology of endothelial cells (cobblestone appearance), presence of CD31 antigen by immunohistochemistry (IHC) (Tachezy et al., 2010) and FITC conjugated Ulex europaeus I lectin antigen (Holthöfer et al., 1982). These antigens are specific for endothelial cells.

### Capillary Tube-Formation Assay

Human brain micro endothelial cells were plated on growth factor reduced basal matrix extract (Corning^TM^ Basal matrix extract^TM^ Membrane Matrix GFR, Cat # CB-40230, Fisher Scientific, Hampton, NH, United States). Tube formation was complete by 16–24 h.

### Cell Migration by Scratch Assay

Cell migration was examined by conducting scratch assay using standard published protocol (Liang et al., 2007). Briefly, 6-well plates were coated with 2% gelatin and then 100,000 HBMECs were plated per well. One microliter/ml of Hoechst 33342 was added to each well for easy cell monitoring. After 24 h the cells were observed for confluency and using a p20 pipet tip a scratch was created to remove the cells from middle of the well. Media was changed to remove the dislodged cells and then images were taken every 6 h using Evos Fluorescence microscope (Thermo Fisher Scientific, Carlsbad, CA, United States). Image analysis was conducted using ImageJ software (Rueden et al., 2017).

### Genome-Wide Transcriptomic Analysis

The microarray was conducted on the biological replicates of six for each group after 24 h of plating of HBMEC cells on Basal matrix extract. We have described these methods in our previous publications (Goyal et al., 2010, 2013; Goyal and Longo, 2012, 2014). Briefly, microarrays for human gene expression analysis were obtained from Agilent Technologies (Santa Clara, CA, United States). The Array ID was GE 8 × 60K V2 and design ID was 039494. Microarray hybridization and scanning were conducted by utilizing commercial services from GenUs Biosystems (Northbrook, IL, United States). Trizol method was used to isolate RNA. RNA purity was assessed by measuring the absorbance at OD260/280, and the RNA with value between 1.8 and 2.0 were included in the study. The RNA quality and fragmentation were examined using an Agilent Bioanalyzer (Supplementary Figure S1). cRNA was prepared using standard techniques as described previously (Goyal et al., 2010, 2013; Goyal and Longo, 2012, 2014). Agilent Bioanalyzer was used to determine the quantity and quality of the labeled cRNA. Before hybridization, 1 μg of purified cRNA was fragmented to a uniform size and then applied to the microarray chips. Microarray chips baked for 17 h at 65°C in a shaking incubator. Following hybridization, the chips were washed at 37°C for 1 min. The chips were dried and then scanned at 5 μm resolution with an G2565 Microarray Scanner from Agilent Technologies. Scanned images from the arrays were processed to conduct gridding and intensity extraction by Feature Extraction software from Agilent. The data obtained was further analyzed by using GeneSpring GX v7.3.1 software (Agilent Technologies). Supplementary Figure S2 demonstrates the probes with intensity above background in at least four replicates (21,221–21,938 probes). Cluster Tree Analysis was conducted to examine the successful clustering of samples based on the sample type (control versus capillary tube formation). This was examined by plotting the fold-change value of differentially expressed genes (>twofold, *p*-value < 0.05), which were normalized to the median expression across the 8 samples (Supplementary Figure S3). The raw data have been deposited at Gene Expression Omnibus (GSE140880).

Gene network, functional pathways, and canonical pathways represented by the differentially expressed genes were analyzed by Ingenuity Pathway Analysis Program (IPA; Ingenuity Systems, Redwood City, CA, United States). An increase (activation) or a decrease (inactivation) in downstream biological activities occurring in the tissues being studied were also analyzed by IPA. Fisher’s Exact Test was used to determine significance (*p*−values < 0.01). A score (*z*−score) was assigned by the IPA software based on literature-derived prediction of “activation” or “inhibition.” The details of the statistics are provided on the IPA website.

### Genome-Wide Methylation Analysis

We have described this method in our previous publication (Mata-Greenwood et al., 2017). Briefly, we isolated the DNA using a chloroform-based extraction method, which was then processed using the EZ DNA Methylation Kit D5008 (Zymo Research, Orange, CA, United States) following the kit’s protocol. Whole-genome methylation analysis was conducted using an Infinium Human Methylation 450K Bead Chip Array from Illumina using the manufacturer’s instruction. Illumina Bead Array Reader was used to image the array and the images were processed to extract intensity data. On the Illumina chip, at each spot, two specific oligomers represent a CpG site. One represents unmethylated DNA (U) and the other represent methylated DNA (M). Intensity values of the M and U alleles, which are differentially colored then represent the fraction of methylated versus unmethylated DNA. This is calculated using the formula ß = [M/(M + U + 100)]. The obtained ß-values were continuous variables between 0 (absent methylation) and 1 (completely methylated) representing the fraction of combined locus intensity. Cluster analysis was conducted by examining hierarchical clustering and principal component analysis.

### Clustered Regularly Interspaced Short Palindromic Repeats Type II System (CRISPR/Cas9)-Mediated Gene Editing

Gene silencing experiments were conducted using CRISPR/Cas9 technology as described by us previously (Mata-Greenwood et al., 2017). We generated lentivirus using third-generation packaging plasmids to conduct gene knockdown experiments. We chose these 11 genes based on the changes in DNA methylation and mRNA expression with the angiogenic transformation of HBMECs for functional validation. The sequences of gRNA used to target the 11 genes and the primers to validate successful upregulation with tube formation and knockdown following CRISPR/Cas9-mediated targeting are provided in Supplementary Table S1. To confirm the successful knockdown of the genes, we conducted quantitative real-time PCR. Primer 3 software was used to design primers and the primers were synthesized by Integrated DNA Technologies (Coralville, CA). cDNA was prepared by using 1 μg total RNA (per reaction) using QuantiTect Reverse Transcriptase Kit (Qiagen, Valencia, CA, United States). Relative gene expression was normalized to the housekeeping gene 18S RNA, and ΔΔCt method was used to determine fold-changes as described previously (Ramakers et al., 2003). Samples were analyzed on the Bio-Rad IQ5 machine.

### Statistics

For all the experiments “*n*” was six in each group. Intensity values obtained from the microarray were normalized to the 75th percentile intensity of each array. Genes were than pre-filtered for four or more samples above background in at least one condition (control or tube formation). Following filtration, the differentially expressed genes were identified by twofold change and Welch *T*-test *p*-values < 0.05. Student’s *T*-test was used to determine the statistical significance of the real-time PCR data. Statistical program “R” was used for hierarchical clustering and principal component analysis.

## Results

### Quality Check

Supplementary Figure S1 demonstrates the gel picture obtained from Agilent Bioanalyzer. As shown in the figure, the 28S and 18S ribosomal bands are visible indicating high quality. The RNA integrity number of all the samples were >8. To further examine the successful clustering of control versus capillary tube formation samples, differentially expressed genes (>twofold, *p*-value < 0.05) were normalized to the median expression across the 12 samples, and Scatter Plot Analysis was conducted. Supplementary Figure S2 demonstrates the Scatter Plot Analysis of the probes which demonstrated intensity above background in six replicates following tube formation. To further examine the differential gene expression in the two groups, the heat map of hierarchical clustering was plotted. As shown in Supplementary Figure S3, there was a significant difference in the gene expression in the two groups examined. Similarly, to demonstrate the successful clustering of samples (control versus capillary tube formation) based on differentially methylated CpG (|β| ≥ ±0.15 and *p*-value < 0.05, *n* = 6 in each group, heat map of hierarchical clustering (Supplementary Figure S4) and principal component analysis (Supplementary Figure S5) was conducted. Overall, the results demonstrate the quality checks were successful for the experiments conducted.

### Genes Altered With Capillary Tube Formation

**FIGURE 1:**
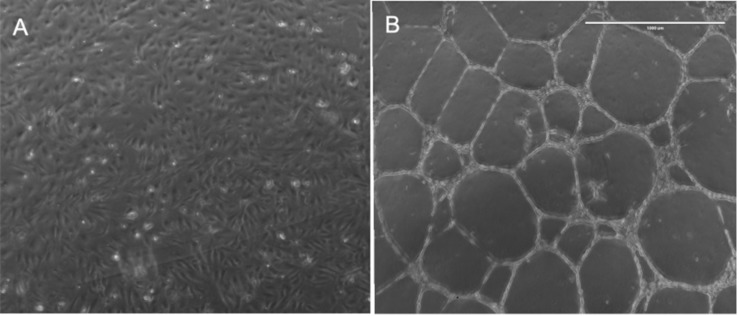
Demonstrates capillary tube transformation of HBMECs following plating on basal matrix extract (BME). Panel **(A)** demonstrates HBMECs 24 h after plating in a well of six-well plate without BME and panel **(B)** demonstrate HBMECs 24 h after plating in a well with BME.

Basal matrix extract-induced transformation of HBMEC cells (Figure 1) was associated with significantly altered expression of 2828 protein-coding genes. Of these, 1318 genes were significantly upregulated (>twofold; *p* < 0.05) and 1490 genes were significantly downregulated (<twofold; *p* < 0.05). A complete list of the upregulated and downregulated genes is provided in the Supplementary Table S2. We compared our HBMEC gene expression data with another microarray study on HUVEC cells tube formation on basal matrix extract (25 h exposure). We observed 39 genes were commonly upregulated and 19 genes were down-regulated (Supplementary Table S3) in both the studies.

### HBMEC Capillary-Tube Formation and Whole-Genome DNA Methylation Analysis

HBMEC tube formation was associated with significant alteration in genome-wide methylation levels. We used the 450K DNA methylation array (Infinium Human Methylation 450 Bead Chip, Illumina Inc., San Diego, CA, United States), which includes a total of 485,764 cytosine sites of the human genome. These cytosine positions include 482,421 CpG sites (99.3%) and 3343 (0.7%) of CNG sites (where *N* is any other nucleotide). Of 482,421 CpG sites examined in the present study, 25,184 showed a significant difference (*p* < 0.05, *n* = 6 in each group) in methylation levels between the control and tube formation group (Supplementary Table S4). Of these 25,184 sites, 8999 CpG were associated with the genes with altered expression. Of these, 4186 CpG sites were in the promoter (Supplementary Table S5). We examined the methylation status of the CpG into north shore, north shelve, island, south shore, south shelve, and open sea (body). CpG Islands were the regions with at least 200 bp with a high frequency of CG dinucleotides (Antequera and Bird, 1993; Saxonov et al., 2006). North shore and south shore were defined as 2 kb regions upstream and downstream of CpG islands, respectively (Irizarry et al., 2009). Whereas, the north and south shelves were 2 kb regions upstream to north shore or downstream to the south shore, respectively (Sandoval et al., 2011). The results demonstrated that north shore had 1494, the north shelf had 427, the islands had 2388, south shore had 1221, the south shelf had 360, and gene body had 3108 CpG sites with altered methylation with tube formation. Moreover, the highest percent of CpGs were altered in the shore region (Table 1).

**TABLE 1:** Number of CpG altered in different gene regions.

**CpG classification**	**Present on Illumina chip**	**Altered with tube formation**	**% CpG altered**
Island	150254	2388	1.6
Shore	112072	2715	2.4
Shelf	47161	787	1.7
Open Sea	176277	3108	1.8

### Correlation of Altered DNA Methylation With Changes in mRNA Expression

We correlated the changes in methylation with the expression value of the corresponding genes. A total of 546 genes were associated with significant changes in the methylation levels. Hypomethylation of the CpG sites was associated with upregulation of 515 genes and hypermethylation of the CpG sites was associated with the downregulation of 31 genes (Supplementary Table S6). However, 243 genes had differentially methylated regions (DMR) in the gene body. Of these 243 CpG sites, 52 were in the first exon of the gene. The remaining 43 CpG were in 3′UTR, 66 CpG were in 5′UTR, 148 CpGs were within 1500 bp upstream of TSS, and 46 CpG with DMR were within 200 bp of TSS. On examining the CpG sites based on north shore, north shelve, island, south shore, south shelve, and open sea (body), we observed 171 CpG were in islands, 26 were in north shelves, 100 were in north shores, 20 were in south shelves, 68 were in south shore, 229 were in open sea (Supplementary Table S6). We also conducted pathway analysis on the genes with both altered mRNA expression and DNA methylation. The major pathways which were altered were cellular response to stress, cellular metabolism pathways, programed cell death pathway, signaling by receptor tyrosine kinase, nuclear receptors, and G-protein coupled receptors.

### Major Pathways Affected by HBMEC Tube Formation

**FIGURE 2:**
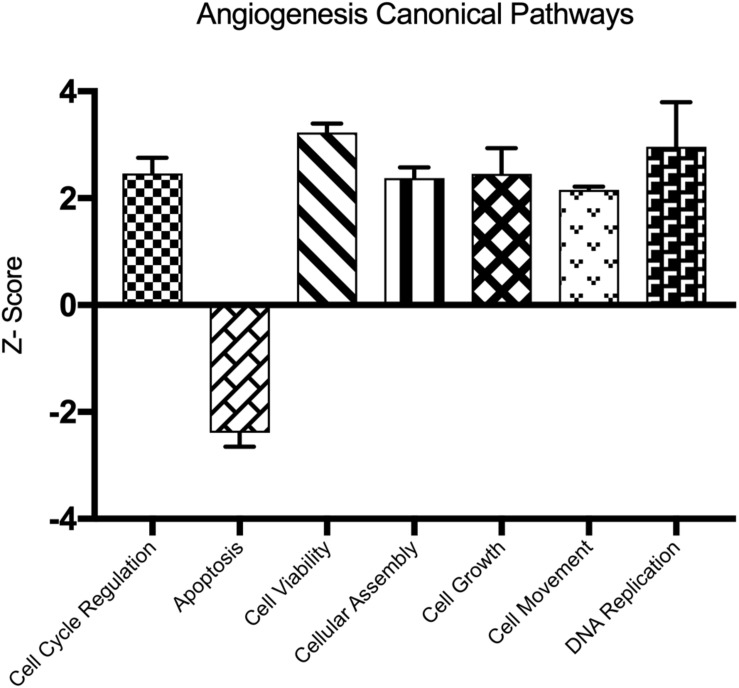
Demonstrates the canonical pathways altered following capillary tube formation identified by Ingenuity Pathway Analysis Software.

We determined important canonical pathway activated following HBMEC tube formation was VDR/RXR activation signaling pathway, and the inactivated pathways were Integrin signaling, interferon signaling, and cAMP-mediated signaling (Table 2). The functional pathways activated were cell cycle, cell survival, cellular assembly, organization, DNA replication, recombination, and repair as well as those involved in the cellular movement (Table 3). The only functional pathway inhibited was the apoptosis pathway (Figure 2).

**TABLE 2:** Canonical pathways altered by capillary tube formation in HBMECs.

**Ingenuity Canonical Pathways**	**−log(*p*-value)**	**Ratio**	***z*-score**
Integrin signaling	1.26	0.164	−2.335
Interferon signaling	1.51	0.25	−2.333
cAMP-mediated signaling	1.05	0.158	−2.121
Cardiac β-adrenergic signaling	1.53	0.185	−2.065
VDR/RXR activation	1.5	0.205	2.449

**TABLE 3:** Functional pathways altered by capillary tube formation.

**Categories**	**Functions**	***p*-value**	**Predicted activation state**	**Activation *z*-score**	**# Molecules**
Cell cycle	Cell cycle progression	1.18E-35	Increased	2.314	350
Cell cycle	Cycling of centrosome	0.00000153	Increased	2.21	29
Cell cycle	Entry into interphase	3.56E-08	Increased	2.872	48
Cell cycle	Entry into S phase	0.00000221	Increased	2.072	41
Cell cycle	Interphase	5.33E-24	Increased	2.407	220
Cell cycle	M phase	1.53E-09	Increased	2.689	74
Cell cycle	S phase	3.6E-13	Increased	2.086	88
Cell death and survival	Apoptosis	0.00000149	Decreased	−2.575	70
Cell death and survival	Cell viability	5.4E-15	Increased	3.105	342
Cell death and survival	Survival	9.22E-17	Increased	3.349	369
Cellular assembly and organization, DNA replication, recombination, and repair	Alignment of chromosomes	2.19E-10	Increased	2.493	18
Cellular assembly and organization, DNA replication, recombination, and repair	Chromosomal congression	5.05E-08	Increased	2.138	10
Cellular assembly and organization, DNA replication, recombination, and repair	Orientation of chromosomes	4.17E-11	Increased	2.493	19
Cellular development, cellular growth and proliferation	Cell proliferation	0.00000441	Increased	2.119	103
Cellular growth and proliferation	Colony formation of cells	7.44E-14	Increased	2.24	151
Cellular growth and proliferation	Proliferation	2.8E-33	Increased	3.01	935
Cellular movement	Cell movement	2.74E-09	Increased	2.112	223
DNA replication, recombination, and repair	DNA replication	0.00000078	Increased	2.709	68
DNA replication, recombination, and repair	Repair of DNA	0.000000147	Increased	3.893	82
DNA replication, recombination, and repair	Synthesis of DNA	2.98E-09	Increased	2.28	114

### Upstream Regulatory Analysis

Using IPA, we found 44 upstream regulatory molecules to be significantly altered with HBMEC angiogenic transformation. Based on the activation prediction, 29 molecules were activated, and 15 molecules were in inhibition state (Table 4). The top upstream regulators identified were CSF2, FOXM1, DDIT3 VEGF, TP53, BNIP3L, and RB1.

**TABLE 4:** Predicted upstream regulators altered with capillary tube formation.

**Upstream regulator**	**Expr. fold change**	**Molecule type**	**Predicted activation state**	**Activation *z*-score**	***p*-value of overlap**
CSF2	9.355	Cytokine	Activated	6.4	3.35E-21
FOXM1	4.077	Transcription regulator	Activated	4.295	3.69E-13
DDIT3	2.445	Transcription regulator	Activated	3.785	0.000704
VEGFA	4.32	Growth factor	Activated	3.27	1.61E-12
ERBB2	2.482	Kinase	Activated	3.05	6.56E-24
BTK	3.33	Kinase	Activated	2.876	0.00162
PARP1	–3.509	Enzyme	Activated	2.786	0.00000626
MITF	–2.132	Transcription regulator	Activated	2.763	1.31E-13
HMGB1	2.045	Transcription regulator	Activated	2.724	0.0667
CREM	2.271	Transcription regulator	Activated	2.624	0.000262
EGR1	8.061	Transcription regulator	Activated	2.577	0.0000507
E2F2	2.911	Transcription regulator	Activated	2.571	8.56E-10
EIF4E	2.695	Translation regulator	Activated	2.549	0.00264
CD24	2.709	Other	Activated	2.495	6.03E-08
SMOC2	2.382	Other	Activated	2.449	0.0000172
MAPK11	–2.625	Kinase	Activated	2.433	0.106
KITLG	4.219	Growth factor	Activated	2.424	0.000654
BMPR2	2.319	Kinase	Activated	2.383	0.00362
IL6	3.91	Cytokine	Activated	2.359	2.55E-12
TRIM24	–2.538	Transcription regulator	Activated	2.267	0.000791
MYCN	–6.897	Transcription regulator	Activated	2.264	0.42
BDKRB2	25.62	G-protein coupled receptor	Activated	2.2	0.0251
IRF6	3.156	Transcription regulator	Activated	2.177	0.0189
MAP2K3	2.89	Kinase	Activated	2.157	0.189
NFATC1	2.523	Transcription regulator	Activated	2.123	0.234
BMP4	–4.854	Growth factor	Activated	2.053	7.04E-08
ATF2	2.195	Transcription regulator	Activated	2.043	0.00977
FLT3LG	–3.04	Cytokine	Activated	2.043	0.44
BMP2	8.23	Growth factor	Activated	2.013	0.0000112
RGS2	3.987	Other	Inhibited	–2.001	0.0444
ATF3	6.606	Transcription regulator	Inhibited	–2.019	0.00277
SMAD1	–3.937	Transcription regulator	Inhibited	–2.02	0.000118
DUSP5	6.956	Phosphatase	Inhibited	–2.121	0.00129
MEF2D	2.209	Transcription regulator	Inhibited	–2.121	0.117
TAZ	–2.123	Enzyme	Inhibited	–2.146	0.00129
ZFP36	2.144	Transcription regulator	Inhibited	–2.598	0.000371
NR4A2	22.09	Ligand-dependent nuclear receptor	Inhibited	–2.619	0.266
RBL1	2.833	Transcription regulator	Inhibited	–2.712	5.89E-08
CTGF	–2.571	Growth factor	Inhibited	–2.763	0.000692
TGM2	–2.004	Enzyme	Inhibited	–3.224	0.0000181
KDM5B	–6.993	Transcription regulator	Inhibited	–3.374	2.21E-08
RB1	2.832	Transcription regulator	Inhibited	–3.759	2.09E-13
BNIP3L	–2.793	Other	Inhibited	–4.26	9.8E-08
TP53	–2.604	Transcription regulator	Inhibited	–4.477	3.4E-40

### CRISPR/Cas9-Mediated Knockdown of Upregulated Genes and Effect on Cell Migration and Angiogenic Transformation

**FIGURE 3:**
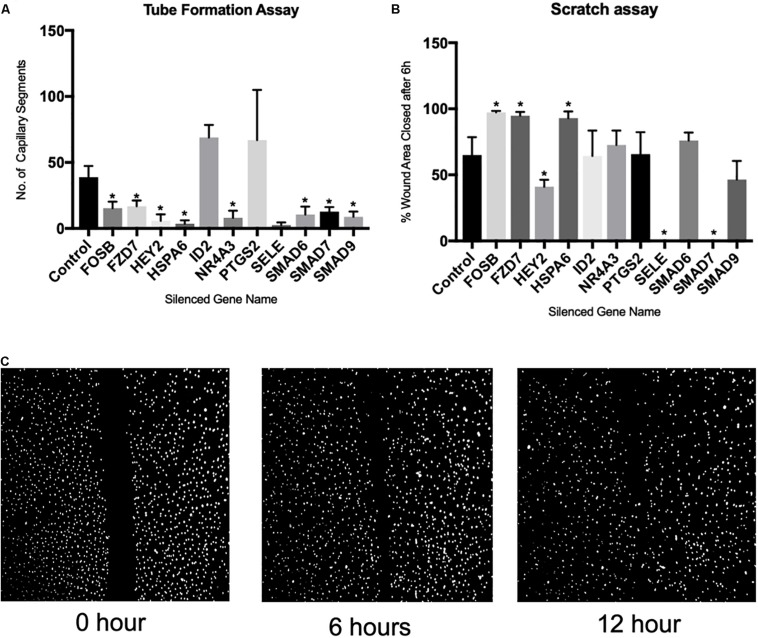
Demonstrates the effect of CRISPR/Cas9-mediated gene silencing on angiogenic process. **(A)** Bar graph showing effect of gene silencing on capillary tube formation, **(B)** bar graph showing effect of gene silencing on cell migration, **(C)** exemplary microscopic images of scratch assay. ^∗^Denotes *p* < 0.05 and *N* = 6 for each assay.

To examine the functional significance of the genes altered as a consequence of capillary tube formation, we generated knockout HBMEC lines of the top 11 genes using CRISPR/Cas9 technology and then conducted tube formation assay on basal matrix extract and cell migration assay (scratch assay). We chose these 11 genes based on their expression changes and correlative methylation alteration. The knockdown of the genes was confirmed by real-time PCR. Following knockdown using CRISPR/Cas9, ID2 and PTGS2 did not show significant alteration in capillary tube formation (Figure 3A). However, there was a significant reduction in capillary tube formation following knockdown of FOSB, FZD7, HEY2, HSPA6, NR4A3, SELE, SMAD6, SMAD7, and SMAD9 (Figure 3A). Of note, the scratch assay (Figure 3C) demonstrated that only HEY2, SELE, AND SMAD7 significantly reduced cell migration (Figure 3B). Interestingly, despite reduced tube formation, FOSB, FZD7, and HSPA6 significantly increased cell migration (Figure 3B).

## Discussion

Angiogenesis is one of the most important processes in healing following the stroke, traumatic brain injury, and other insults leading to hypoxia/ischemia region. It is a vital process in various physiological states such as development, organogenesis, placentation, and especially for the optimal reproductive cycle of women. Angiogenesis is also a critical process in various pathological conditions such as organ repair, cutaneous wound healing, cancer, myocardial infarction, and stroke. However, pathways regulating angiogenesis are not well defined. Specially, there are very few studies on human brain microendothelial cells. In the present study, we investigated the gene expression changes in HBMECs. Additionally, DNA methylation is known to play a significant role in angiogenesis. However, the specific genes with altered methylation levels during the angiogenic transformation of endothelial cells are not known. In the present study, we demonstrate that hypomethylation of the promoter region as the major process during angiogenic transformation. This also validates the recent finding that loss of methyl-CpG-binding domain protein 2 enhances endothelial angiogenesis (Rao et al., 2011).

Functional validation of FOSB, FZD7, HSPA6, HEY2, SMAD6, SMAD7, SMAD9, NR4A3, and SELE by CRISPR/Cas9 knockdown demonstrated significant inhibition of capillary tube transformation of HBMEC cells. Of note, most of the genes with significant alterations in expression have changes in DNA methylation levels. Thus, the present study provides a comprehensive list of molecules for further investigations to determine the functional pathways of angiogenesis.

The study provides several novel molecules whose role in angiogenesis have not well studied. For instance, the role of Hairy and Enhancer-of-split-related basic helix-loop-helix (bHLH) transcription factors is not well defined in angiogenesis *per se*. However, expression of HEY family gene is induced by the Notch and c-Jun signal transduction pathways and are known to play an essential role during development (Fischer et al., 2004). We observed several members of this group of transcription factors upregulated with altered promoter methylation following angiogenic transformation.

Similarly, little is known regarding the role of NR4A3 receptors in angiogenesis. However, NR4A1 and 2 are known to be the downstream targets of VEGF, promoting the proliferation of endothelial cells *in vitro* and *in vivo* (Zhao et al., 2011). The present study demonstrates that NR4A3 inhibition significantly inhibited HBMEC angiogenic transformation and may be used to target brain tumors. Downregulation of NR4A3 significantly inhibited HBMEC capillary tube formation.

Similarly, the SMAD pathway is a canonical signaling pathway of the TGF-β family members, which is known to have a significant role in angiogenesis. The SMAD family member proteins SMAD6, SMAD7, SMAD9 (fold upregulated 31.87, 11.54, 9.41, respectively) play a significant role during angiogenic transformation. SMADs are intracellular proteins and transcription factors that transfer extracellular signals from growth factor ligands to the nucleus to activate downstream gene transcription (Heldin et al., 1997; Attisano and Wrana, 2002). Moreover, knockdown of SMAD6, 7, and 9 by CRISPR/Cas9 leads to significant inhibition of angiogenic transformation of HBMEC cells. Similarly, a recent report demonstrated that SMAD knockdown leads to inhibition of angiogenesis on collagen gel model (Lin et al., 2016).

On examining gene knockdown studies in mice, Hey1 and Hey2 combined gene knockdown is embryonic lethal with a global lack of vascular development (Fischer et al., 2004). Similarly, a recent study demonstrated that endothelial cell deletion of FZD7 delayed retinal plexus formation and postnatal angiogenesis (Peghaire et al., 2016). Both of these genes belong to the Notch signaling pathway.

## Conclusion

Angiogenic transformation altered the expression of hundreds of genes which may be regulated by underlying epigenetic changes such as DNA methylation. The present study provides a number of genes with altered methylation in various parts of the genes in brain endothelial cells. The functional, canonical, and upstream pathways identified by the present studies requires further investigation, however.

## Data Availability Statement

The datasets generated for this study can be found in the Gene Expression Omnibus (GEO) Database Accession No. GSE140880.

## Author Contributions

The study was designed by RG. Data collection and analysis were conducted by DG. RG and DG wrote the manuscript.

## Conflict of Interest

The authors declare that the research was conducted in the absence of any commercial or financial relationships that could be construed as a potential conflict of interest.

## Supplementary Material

FIGURE S1 Demonstrates quality control gel image of total RNA isolated from 6 controls (untreated HBMECs) and 6 transformed HBMECs.

FIGURE S2 Demonstrates the Scatter Plot Analysis of the probes which demonstrated intensity above background in six replicates following tube formation as compared to control HBMECs.

FIGURE S3 Demonstrates a heat map of hierarchical gene cluster analysis of the significantly altered (*p* < 0.05) mRNA from 6 transformed HBMECs as compared to control.

FIGURE S4 Demonstrates a heat map of hierarchical gene cluster analysis of the significantly altered (*p* < 0.05) methylation CpG regions in the genes from 6 transformed HBMECs as compared to control.

FIGURE S5 Demonstrates Principal Component Analysis to show the successful clustering of samples (control versus capillary tube formation) based on differentially methylated CpG (| β| ≥ ±0.15 and *p*-value < 0.05, *n* = 6 in each group.

TABLE S1 List of genes silenced by CRISPR/Cas9 with gRNA sequences and primers to validate knockdown. Also enumerated are the effect of knockdown on capillary tube formation and cell migration.

TABLE S2 Enlists the upregulated and downregulated genes (>twofold; *p* < 0.05) as a result of capillary tube formation of HBMECs.

TABLE S3 Enlists genes commonly altered as a result of capillary tube formation in the present study conducted on HBMECs and a similar study (GSE7507) conducted on HUVECs.

TABLE S4 Enlists the CpG sites with significantly altered (*p* < 0.05) methylation as a result of capillary tube formation.

TABLE S5 Enlists genes with change in CpG methylation in promoter region with correlated change in mRNA levels.

TABLE S6 Enlists genes with correlated changes in mRNA expression and CpG methylation.
