# Supplementary figures and images for: Angiogenic Transformation in Human Brain Micro Endothelial Cells: Whole Genome DNA Methylation and Transcriptomic Analysis
Source: Front Physiol. 2019 Dec 11;10:1502. doi: 10.3389/fphys.2019.01502 (PMC6917667; doi:10.3389/fphys.2019.01502)

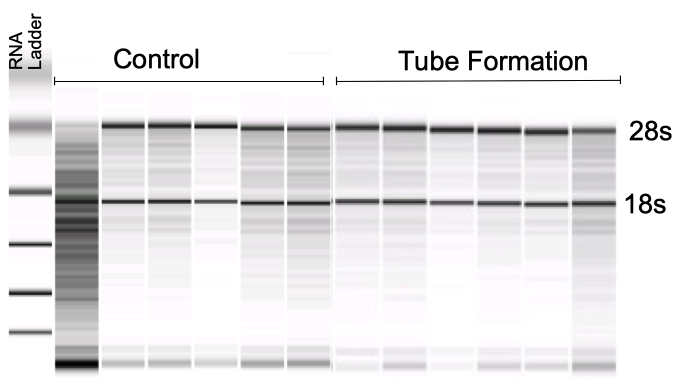

Supplement: FIGURE S1 — Demonstrates quality control gel image of total RNA isolated from 6 controls (untreated HBMECs) and 6 transformed HBMECs. [file Image_1.TIF]

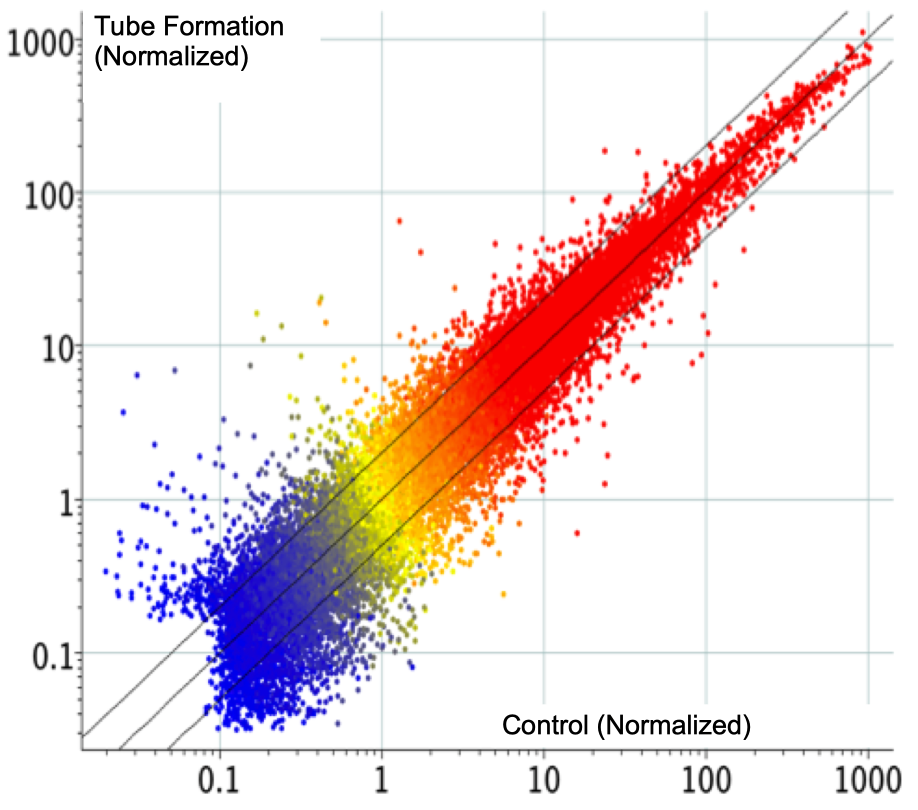

Supplement: FIGURE S2 — Demonstrates the Scatter Plot Analysis of the probes which demonstrated intensity above background in six replicates following tube formation as compared to control HBMECs. [file Image_2.TIF]

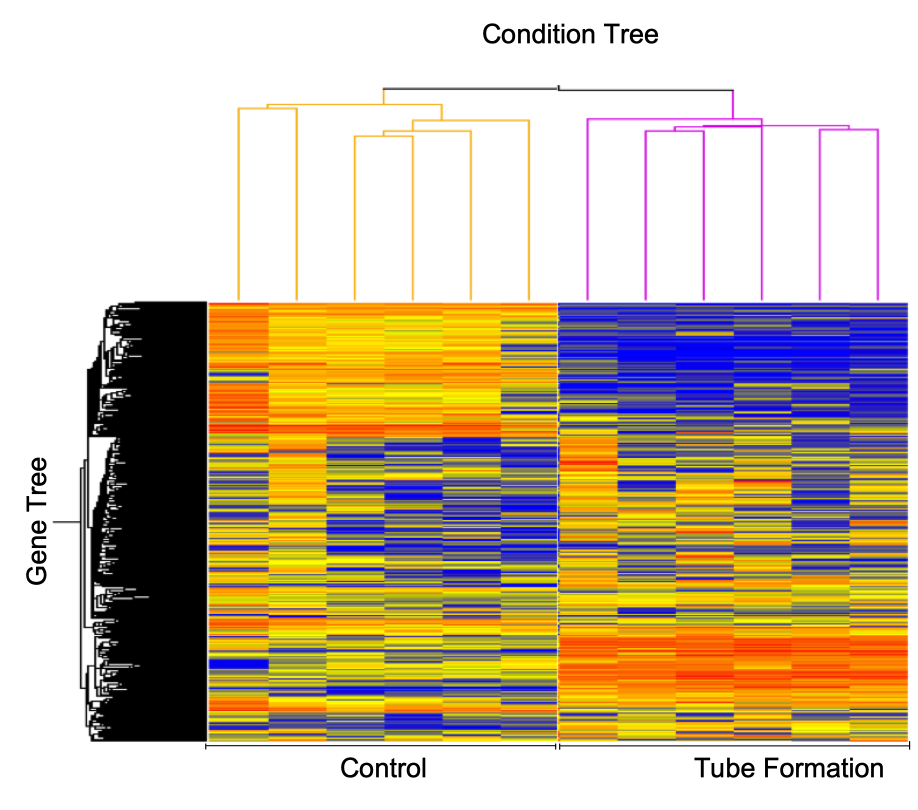

Supplement: FIGURE S3 — Demonstrates a heat map of hierarchical gene cluster analysis of the significantly altered (p < 0.05) mRNA from 6 transformed HBMECs as compared to control. [file Image_3.TIF]

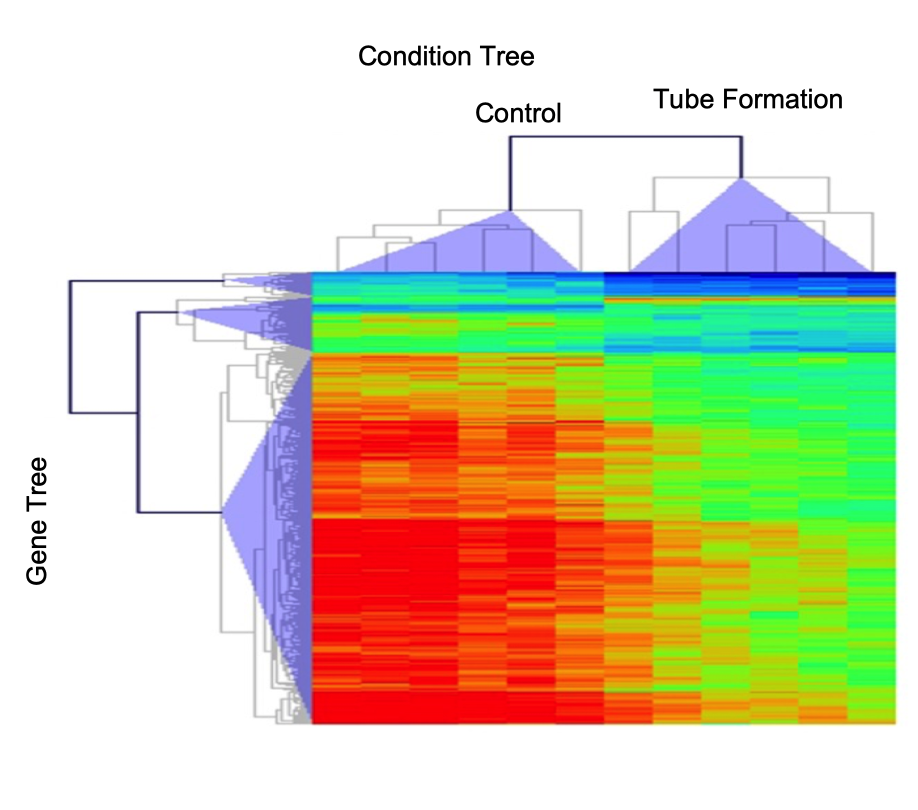

Supplement: FIGURE S4 — Demonstrates a heat map of hierarchical gene cluster analysis of the significantly altered (p < 0.05) methylation CpG regions in the genes from 6 transformed HBMECs as compared to control. [file Image_4.TIF]

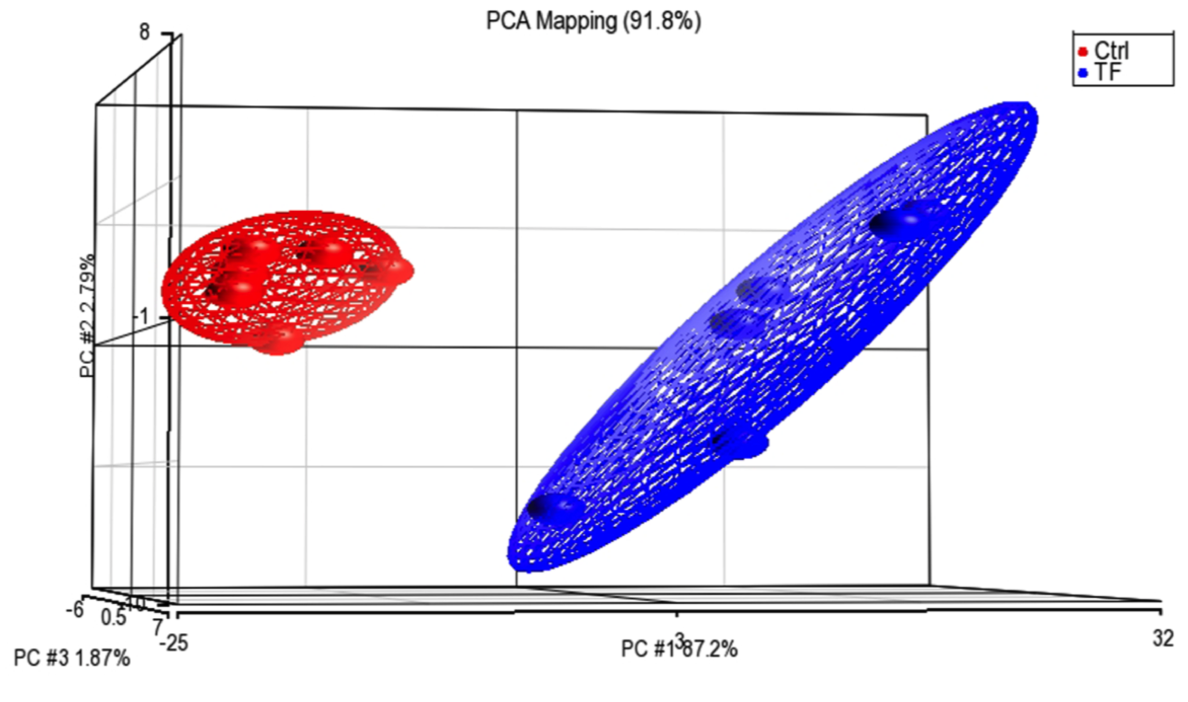

Supplement: FIGURE S5 — Demonstrates Principal Component Analysis to show the successful clustering of samples (control versus capillary tube formation) based on differentially methylated CpG (| β| ≥ ±0.15 and p-value < 0.05, n = 6 in each group. [file Image_5.TIF]
